# Smallholder Cattle Farmers’ Knowledge, Attitudes, and Practices Toward Rabies: A Regional Survey in Kazakhstan

**DOI:** 10.3390/vetsci12040335

**Published:** 2025-04-04

**Authors:** Nurbek Ginayatov, Zukhra Aitpayeva, Izimgali Zhubantayev, Leila Kassymbekova, Assylbek Zhanabayev, Gulmira Abulgazimova, Raikhan Arynova, Alim Bizhanov, Assiya Mussayeva, Maxat Berdikulov, Marat Aisin, Zaure Sayakova, Spandiyar Tursunkulov, Nurkuisa Rametov, Ainur Akhmadiyeva, Aigul Bulasheva, Nurgul Jussupbekova, Olzhas Yeskhojayev, Gulnara Baikadamova, Kaissar Kushaliyev, Nadezhda Burambayeva, Arman Issimov

**Affiliations:** 1 Laboratory of Biotechnology and Diagnosis of Infectious Diseases, Zhangir KhanWest Kazakhstan Agrarian-Technical University, Oral 030000, Kazakhstan; 2 Department of Veterinary Medicine, Zhangir KhanWest Kazakhstan Agrarian-Technical University, Oral 030000, Kazakhstan; 3 Department of Veterinary Medicine and Industrial Technologies, Innovative University of Eurasia, Pavlodar 140000, Kazakhstan; 4 Department of Veterinary Medicine, Saken Seifullin Kazakh Agrotechnical University, Astana 010000, Kazakhstan; 5 Department of Hunting and Fisheries, Saken Seifullin Kazakh Agrotechnical University, Astana 010000, Kazakhstan; 6 Department of Bacteriology, Kazakh Research Veterinary Institute, Almaty 050016, Kazakhstan; 7 Department of Veterinary Medicine, A. Baitursynov Kostanay Regional University, Kostanay 110000, Kazakhstan; 8 Department of Geospatial Data Science, Institute of Ionosphere, Almaty 050016, Kazakhstan; 9 Department of Veterinary Medicine, Shakarim University, Semey 071412, Kazakhstan; 10 Department of Parasitology, Institute of Zoology, Almaty 050016, Kazakhstan; 11 Department of Plague Microbiology and Epidemiology, Masgut Aikimbayev’s National Scientific Center for Especially Dangerous Infections, Almaty 050016, Kazakhstan; 12 Department of Zootechnology, Genetics and Breeding, Toraighyrov University, Pavlodar 140000, Kazakhstan; 13 Department of Biology, K. ZhubanovAktobe Regional University, Aktobe 030000, Kazakhstan

**Keywords:** rabies, knowledge, attitudes, practices, Kazakhstan, rabies prevention

## Abstract

Rabies remains a significant public health and economic concern in Kazakhstan, particularly in rural livestock-farming communities. This study aimed to assess the knowledge, attitudes, and practices (KAPs) related to rabies among livestock farmers in the Aktobe and Oral regions of West Kazakhstan. A cross-sectional survey was conducted between April and August 2022, involving 688 randomly selected participants. The data were collected through structured interviews and analyzed using descriptive and inferential statistics. The findings revealed that 89% of respondents were aware of rabies, yet significant knowledge gaps existed regarding clinical signs, transmission, and prevention. While 87% recognized the importance of rabies vaccination in dogs, 81% were unaware of pre-exposure prophylaxis (PrEP) for cattle, and 72% lacked knowledge of PrEP for humans. Awareness of the post-exposure prophylaxis (PEP) regimen was significantly higher in the Aktobe region (*p* < 0.002). Attitudinal differences were observed, with the Oral region participants exhibiting more favorable perceptions of rabies control programs (*p* < 0.01). Additionally, the χ^2^ test revealed that the proportion of female respondents (*p* < 0.02), those with school-aged dependents (*p* < 0.003), respondents owning both exotic and indigenous cattle breeds (*p* < 0.002), and those possessing more than five cattle (*p* < 0.025) was statistically different in the Oral region. Practices such as free grazing, lack of protective equipment use, and improper carcass disposal were identified as potential risk factors for rabies transmission. This study highlights the need for targeted educational initiatives to improve rabies awareness and promote safer livestock management practices. Enhancing veterinary surveillance, strengthening community engagement, and expanding vaccination efforts could mitigate rabies transmission risks.

## Simple Summary

Rabies is a deadly disease threatening both humans and animals, especially in rural farming areas. This study surveyed over 600 farmers in Kazakhstan to understand their knowledge and behaviors regarding rabies. While most farmers knew about rabies, many were unaware of key details such as how it spreads and how to prevent it. Risky practices like free grazing and unsafe handling of animal remains were common. However, many farmers showed readiness to improve if given the proper guidance. These findings are important for planning better rabies education and control programs in Kazakhstan.

## 1. Introduction

Rabies is a contagious and life-threatening viral zoonotic disease caused by the rabies virus, a member of the *Lyssavirus* genus within the *Rhabdoviridae* family. Globally, the virus is responsible for over 60,000 human fatalities each year, with 40% being children under 15 in Africa and Asia [1]. However, the actual mortality burden is estimated to be as high as 100,000 deaths annually. Additionally, it is reported that for each documented case of rabies, up to 10 cases may remain unreported [2]. Domestic dogs serve as the primary vectors for the transmission of rabies to humans, a disease that is preventable through the use of personal protective equipment (PPE) [3]. However, access to PPE remains limited in many developing countries [4]. The control and eventual eradication of human rabies largely depends on the effective management and reduction of rabies within canine populations. Central Asian countries can be classified based on their rabies status into four categories: low-risk, medium-risk, high-risk, and rabies-free regions. In Kazakhstan, rabies is an endemic disease, primarily affecting domestic and wildlife animals. However, occasional human cases have been reported annually. According to the World Health Organization, 51 rabies-related human deaths were registered in Kazakhstan between 2010 and 2022 [5].

Kazakhstan, the ninth-largest country in the world by land area, encompasses a geographically diverse landscape where rabies cases have historically been documented across a range of domestic and wildlife species [6]. The animals most frequently implicated in reported rabies cases include cattle, dogs, foxes, sheep, goats, cats, horses, camels, wolves, and jackals. Among these, cattle have consistently demonstrated the highest annual incidence of rabies, imposing a significant economic burden [7]. According to the Global Alliance for Rabies Control, dog rabies is endemic in Kazakhstan, with an annual vaccination coverage of 21.10%. Additionally, no in-country Stepwise Approach towards Rabies Elimination (SARE) assessment has been conducted [8]. The lack of structured surveillance and reporting mechanisms hinders the ability to accurately assess **positive cases, the effectiveness of prevention programs, and the risk of interspecies transmission,** particularly between **infected wildlife, stray dogs, and livestock.** Consequently, the true burden of bovine rabies in Kazakhstan is likely underestimated, emphasizing the need for **enhanced monitoring and data collection efforts** to inform targeted prevention strategies. Defining the knowledge, attitudes, and practices (KAPs) of livestock owners regarding rabies is essential for developing effective strategies to prevent rabies outbreaks in cattle and mitigate the associated public health and economic burdens. Previous retrospective studies identified notable spatial and temporal clustering of rabies cases in Kazakhstan [9,10]. However, to the best of our knowledge, no KAP studies regarding rabies have been previously conducted in Kazakhstan. This study aims to evaluate KAPs regarding rabies among livestock farmers in selected areas of West Kazakhstan.

## 2. Materials and Methods

### 2.1. Study Area

**Figure 1:**
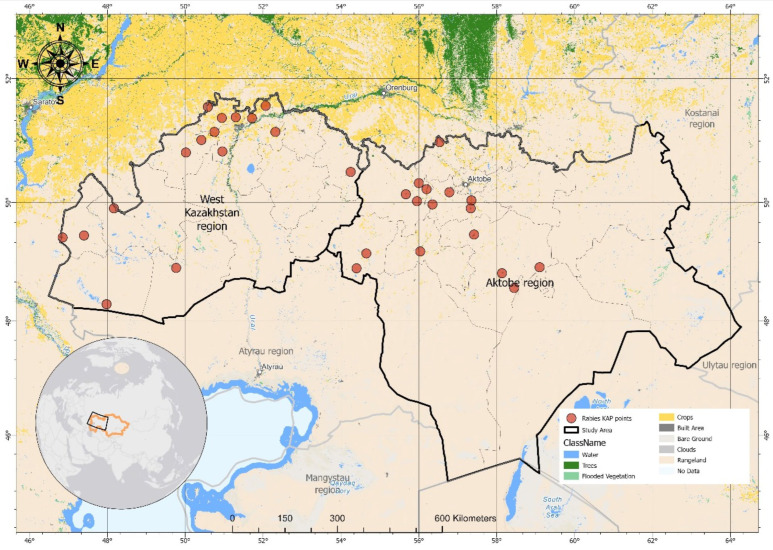
Selected districts of West Kazakhstan region for rabies KAP study. The black-marked region highlights cattle distribution density, which is a key factor in understanding rabies transmission risks. Areas with higher cattle populations may have increased livestock exposure to rabid wildlife or stray dogs, necessitating enhanced surveillance and vaccination efforts. Cattle population data were sourced from the Food and Agriculture Organization [15].

The present study was conducted between April and August 2022 in the rural areas of the Aktobe and Oral regions in West Kazakhstan (51.7758986′ N, 64.2771740′ E). This region covers an area of 736,241 km^2^ and has a population of about 3.1 million people and 2.2 million livestock [11]. These regions were selected due to the high concentration of livestock [12] and documented rabies cases among agricultural, domestic, and wildlife species between 2003 and 2014 [9]. The map was developed using ArcGIS Pro 2.8 (ESRI, Redlands, CA, USA), a geographic information system (GIS) software used for spatial data visualization and mapping (Figure 1). The geographical data were projected using the **World Geodetic System 1984 (WGS),** a standard global reference framework for latitude and longitude coordinates [13]. Field data were imported and integrated with ancillary datasets, including satellite imagery (Sentinel-2 10 m, Land Use/Land Cover), topographic layers, and municipal boundaries [14].

### 2.2. Sample Size

The sample size determination was based on the method described by Thrusfield [16]. To do so, an anticipated prevalence of 50%, a 95% confidence level, and a precision of 5% were utilized to collect adequate data on the KAPs related to rabies among farmers in selected districts. The calculated sample size for the study was 454 participants. However, for the consideration of potential clustering effects and non-response rates, an additional 50% of the sampling units were included, resulting in a total sample size of 688 participants. Similar approaches have been employed in rabies KAP studies in Ethiopia [17] and Uganda [18], demonstrating their effectiveness in capturing reliable data in rural livestock-owning populations. This approach ensured that our study was adequately powered to detect statistically significant differences in rabies KAPs among farmers in the selected regions. Official records of livestock owners in the areas studied were obtained from the District Veterinary Office (DVO). Sample size was selected randomly utilizing Microsoft Excel software by assigning a random number to each farm owner. Farmers were surveyed based on their willingness to participate in the study. In cases where a selected farmer declined to participate, an alternative farmer was chosen as a replacement. The overall participation rate was 90%. All interviewers were trained in administering the survey.

### 2.3. Questionnaire

A structured questionnaire consisting of three sections was developed and utilized for data collection (Table S1). The first section included questions designed to capture participants’ socio-demographic information. Sections two and three focused on the key questions assessing rabies knowledge, including the recognition of clinical signs, transmission routes, and preventive measures such as vaccination. Attitudinal questions focused on the perceptions of rabies risk and willingness to support control programs, while practice-related questions examined behaviors such as wound treatment, livestock handling, and reporting suspected cases, as described in the literature [19]. All questions in these four sections were formatted as closed-ended. The questionnaire was piloted and subsequently revised after five initial interviews to enhance clarity and refine questions. Face–to-face interviews with farm owners were conducted in either Kazakh or Russian, based on the language preference of the participants.

### 2.4. Statistical Analysis

The data were collected into a Microsoft Excel spreadsheet (Table S2), edited, and processed using the Python programming language (Jupyter Notebook, version 3.9.7). Descriptive and univariate analyses were conducted to quantify respondents’ KAPs regarding rabies, and results were shown as percentages. A value of *p* ≤ 0.05 was designated statistically significant. A frequency table was utilized to present participants’ awareness, knowledge, and educational levels in terms of absolute numbers and percentages. Respondents were categorized based on their educational background to compare and evaluate their baseline knowledge and awareness of rabies. Each respondent’s educational level and corresponding awareness was displayed in absolute numbers and percentages. The results from the final models are presented as odds ratios with corresponding 95% confidence intervals. The chi-square test was used to determine associations between categorical variables, particularly to compare differences in rabies knowledge, attitudes, and practices between study groups. Multivariable regression analysis was applied to control for potential confounders and assess the independent effects of demographic and behavioral factors on KAP outcomes. These methods were chosen to enhance the robustness and interpretability of the findings. Participants were categorized into levels of good/poor knowledge, positive/negative attitudes, and appropriate/inappropriate practices based on their respective percentages. The variables associated with rabies incidence were shortlisted for consideration in the final multivariate logistic regression. The pair-wise interaction test was used to define the effects of interactions between all factors in the final multivariable logistic model.

## 3. Results

### 3.1. Socio-Demographic Characteristics

A total of 688 participants were interviewed, with 344 respondents from the Aktobe region and 344 from the Oral region. The mean age of participants did not differ (*p* = 0.33) between the Aktobe (52.2 years) and Oral (53.7 years) regions. The χ^2^ test revealed that the proportion of female respondents (*p* < 0.02), those with school-aged dependents (*p* < 0.003), respondents owning both exotic and indigenous cattle breeds (*p* < 0.002), and those possessing more than five cattle (*p* < 0.025) was statistically different in the Oral region (Table 1).

### 3.2. Knowledge of Respondents Regarding Rabies

**Figure 2:**
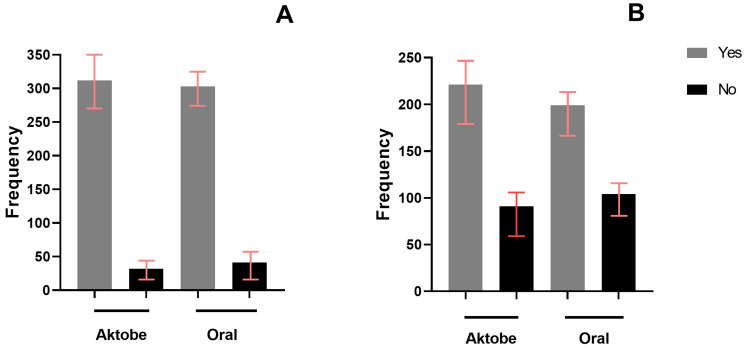
Distribution of participants who were aware of rabies (**A**) and had observed a rabies case in either dogs or livestock (**B**), classified by study regions (n = 688).

Overall, 615 out of 688 participants (89%) were aware of rabies (Figure 2A). In the Aktobe region, 312 participants (91%) reported awareness of rabies, compared to 303 participants (88%) in the Oral region. Awareness of rabies was not significantly correlated (r = 0.38, *p* = 0.902) with the participants’ residency. Among those who were aware of rabies, 166 individuals (27%) were unaware of the clinical signs exhibited by a rabid dog, and 252 participants (41%) indicated that they would be unable to recognize rabies in cattle. Although respondents from the Oral region demonstrated significantly lower awareness of how rabies manifests in dogs (*p* < 0.013), no difference was observed between the two regions in the proportion of respondents unable to identify rabies in cattle, with 168 (54%) in the Aktobe region and 176 (57%) in the Oral region. Among those aware of rabies, a total of 368 participants (60%) had encountered a rabies case, whether in dogs or livestock. The dwelling location was not significantly correlated with having witnessed a rabies case. In the Aktobe region, 221 participants (71%) had seen a rabies case, compared to 199 participants (66%) in the Oral region (Figure 2B).

In this study, a vast majority of participants 535 (87%) were aware of preventive rabies vaccination for dogs. No significant correlation (r = 0.40, *p* = 0.955) was observed between awareness and place of residence. Overall, 498 participants (81%, *n* = 615) were unaware of pre-exposure prophylactic vaccination (PrEP) for cattle. In the Aktobe region, 206 participants (66%, *n* = 312) reported not knowing about PrEP in cattle, compared to 236 participants (78%, *n* = 303) in the Oral region. Additionally, 443 interviewees (72%, *n* = 615) indicated a lack of awareness regarding the availability of PrEP for humans.

A total of 467 participants (72%) acknowledged that rabies is preventable in humans if appropriate measures are taken following potential exposure. Most of them—425 (69%)—were aware of the standard post-exposure prophylaxis (PEP) protocol, which involves disinfecting the bite wound and administering five to six doses of rabies vaccine. Among the remaining participants, 37 (6%) identified wound washing with soap and water as the sole component of the standard PEP regimen, 67 (11%) mentioned wound washing combined with a single vaccine dose, and 86 (14%) reported being unaware of the standard PEP protocol. Awareness of the standard post-exposure prophylaxis (PEP) regimen was significantly higher among participants from the Aktobe region compared to those from the Oral region (*p* < 0.002).

**Figure 3:**
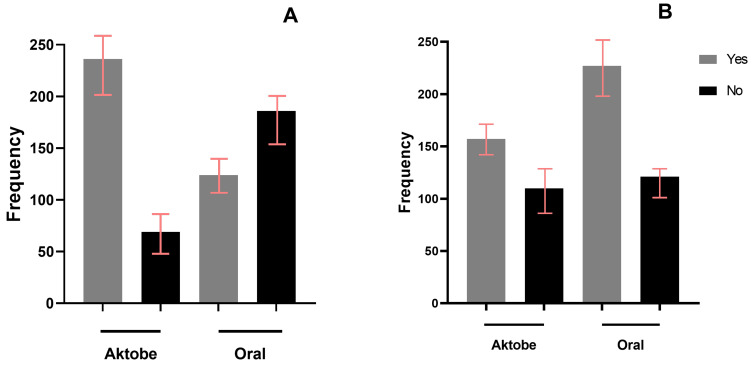
Comparison of participants with adequate/inadequate knowledge of rabies (**A**) and those with favorable/unfavorable attitudes toward rabies prevention and control programs (**B**), classified by study regions (*n* = 615).

Among interviewees who were aware of rabies, 271 individuals (44%) demonstrated sufficient knowledge about the disease (Figure 3A). Multivariable regression analysis revealed that the level of knowledge about rabies was significantly higher in participants residing in the Aktobe region compared to those in the Oral region. The proportion of respondents with an adequate level of knowledge about rabies transmission routes was higher among those from the Aktobe region (47%, *n* = 312) compared to those from the Oral region (35%, *n* = 303). Additionally, participants who had observed a rabies case exhibited significantly greater knowledge of the disease compared to those who had not (Table 2).

### 3.3. Attitude and Practices of Participants Regarding Rabies

Approximately two-thirds of participants 431 (70%) who were aware of rabies exhibited a favorable attitude toward disease prevention and control initiatives (Figure 3B). Regionally, 190 participants (61%) in the Aktobe region demonstrated a favorable attitude, compared to 236 participants (78%) in the Oral region. The multivariable logistic regression analysis indicated that respondents from the Oral region had a greater favorable attitude toward rabies prevention and control programs compared to those in the Aktobe region (Table 3).

A total of 117 participants (17%) reported practicing stall feeding for their cattle. In terms of grazing practices, 172 participants (25%) practiced extensive grazing, 275 (40%) utilized semi-extensive grazing, and 124 (18%) tethered their cattle. Among the 688 respondents, 220 participants (32%) indicated that they did not have a dedicated barn for their cattle, while 310 participants (45%) reported using barns. Nearly one-fourth of participants—158 (23%)—reported having access to reliable cattle barns featuring concreted floors and properly roofed structures. The proportion of participants with reliable cattle barns was higher in the Aktobe region (162 participants, 47%) compared to the Oral region (107 participants, 31%).

Most of the participants—598 (87%)—indicated that they would either report the death of an animal due to illness to a DVO or bury the carcass. Among these, 474 participants (69%) stated that they would bury the carcass, while 165 participants (24%) reported that they would notify a DVO. Conversely, 48 participants (7%) admitted to dressing the carcass for the purpose of selling the meat.

Overall, 578 participants (84%) reported providing assistance to their cattle during parturition, while 550 (80%) reported handling diseased animals. Additionally, 640 participants (93%) indicated examining the oral cavities of their cattle, 544 (79%) reported treating animal wounds, and 213 (31%) acknowledged processing carcasses. The proportion of individuals who were involved in slaughtering animals was twice as high in the Oral region compared to the Aktobe region (41% vs. 19%, respectively). However, across both study areas, 92% of participants who engaged in the specified activities reported practicing handwashing after completing on-farm tasks.

## 4. Discussion

Collecting data on the KAPs of livestock farmers is crucial for the design, implementation, and evaluation of programs aimed at the control and prevention of infectious diseases [20]. To the best of our knowledge, this is the first KAP study examining livestock owners’ perspectives on rabies in selected rural areas of West Kazakhstan. Moreover, no KAP studies or comprehensive rabies epidemiology research has been conducted in neighboring Central Asian countries such as Uzbekistan and Kyrgyzstan. Our literature search did not identify any published studies related to rabies awareness, livestock owner perceptions, or systematic surveillance in these regions. Previous research in Kazakhstan has primarily focused on the epidemiological surveillance and spatial distribution of rabies cases [9]; however, limited studies have examined the human behavioral factors influencing disease transmission and control measures. By identifying critical gaps in knowledge, such as the limited awareness of PrEP for livestock and misconceptions about PEP in humans, this study provides empirical data that can directly inform targeted intervention programs.

The overall awareness of rabies was consistently high across both the Aktobe and Oral study regions, aligning with findings from community-based surveys carried out in rural areas of Southern Ethiopia [21]. Nonetheless, certain knowledge gaps were identified among participants in both areas studied. For example, some respondents were unaware of the appropriate measures to take following potential exposure to rabies. Furthermore, even among those who reported that they would seek medical assistance after exposure, a significant proportion were unaware that the correct post-exposure prophylaxis regimen involves thorough wound washing followed by a series of five to six doses of the rabies vaccine. The lack of awareness regarding the correct PEP regimen among participants could partially explain why some individuals fail to complete the required vaccination course following potential exposure. A previous study investigating the utilization and distribution of human rabies PEP vaccines estimated that approximately 40% of exposed individuals did not adhere to the recommended PEP regimen [2].

As anticipated, a substantial proportion of respondents were unaware of PrEP for humans. This knowledge gap can be attributed to the fact that the existing rabies education initiatives at the community level primarily focus on mass dog vaccination campaigns and promoting appropriate medical interventions following potential exposure. Moreover, according to the WHO, the mass administration of PrEP is recommended only in regions with a high rabies burden and among high-risk occupational groups, such as veterinarians and wildlife handlers [1].

In Kazakhstan, human and livestock vaccinations are predominantly provided by the government [22,23]. However, due to the absence of a formal government policy supporting the implementation of PrEP in livestock species, routine rabies vaccination is not practiced in these animals. Current rabies control initiatives in Kazakhstan are primarily aimed at eradicating canine-associated rabies through mass dog vaccination campaigns. These factors likely contribute to the low level of awareness among study participants regarding rabies PrEP for cattle. To reduce the economic and public health burden of rabies, the World Organization for Animal Health (WOAH) advocates for the vaccination of cattle and other livestock in rabies-endemic regions where the risk of exposure is significant [24]. Mass dog vaccination is widely recognized as a cost-effective and evidence-based strategy for eliminating rabies virus in canine populations, and Kazakhstani government plans to continue implementing mass dog vaccination campaigns in alignment with current policy guidelines [25].

The proportion of respondents with adequate knowledge of rabies was lower than expected based on the study’s predefined criteria. The findings of this study align with previous research conducted in other developing countries such as Tanzania and Ethiopia [17,26]. Studies in Pakistan [19] and Sri Lanka [27] suggested that structured community-based rabies control programs have improved public awareness and vaccination rates. However, our study represents the first assessment of rabies-related knowledge and attitudes among livestock owners in Kazakhstan, revealing areas where knowledge is insufficient or inconsistent, particularly regarding PEP and PrEP. These findings emphasize the need for tailored educational interventions aligned with the local context of Kazakhstan’s livestock sector. These international comparisons highlight a critical point: Kazakhstan’s relatively low scores in certain knowledge areas likely reflect the historic lack of community-focused rabies education, but they also indicate room for improvement if proven strategies from other regions are adapted. Implementing regular village-level trainings or school-based programs, as done in parts of South Asia [28], could similarly elevate rabies knowledge and prevention practices in Kazakhstan.

The multivariable logistic regression analysis revealed that respondents residing in the Aktobe region who observed cases of rabies were positively associated with possessing adequate knowledge about the disease. The higher proportion of participants in the Aktobe region possessing adequate knowledge about rabies underscores the additional focus placed by the Kazakhstani government on awareness campaigns and control programs in high-risk areas with elevated rabies prevalence. The identification of a low percentage of respondents in the Oral region with sufficient knowledge about rabies underscores the necessity of strengthening veterinary surveillance in this region and raising public awareness. This, in turn, could improve reporting rates and enhance vaccination coverage among dogs. The multivariable regression analysis revealed that education level, prior exposure to rabies cases, and access to veterinary services were significant predictors of rabies knowledge. Notably, individuals with higher education levels and previous exposure to rabies demonstrated stronger knowledge retention. Additionally, access to veterinary services not only influenced knowledge but also shaped attitudes toward vaccination and reporting practices. The interplay between these factors underscores the need for an integrated approach that combines public awareness campaigns with improved access to veterinary services to enhance rabies prevention efforts

It was reported that the successful implementation of rabies control initiatives is highly dependent on community engagement [29]. In addition to vaccination, improving cattle management practices, such as reducing free grazing and providing proper housing, can help minimize contact between cattle and stray dogs, thereby preventing potential exposures to rabies. However, our findings indicate that only a small proportion of respondents reported having adequately constructed cattle barns, whereas a vast majority allowed their livestock to graze freely with minimal supervision. Given the traditional extensive and semi-extensive livestock rearing practices, coupled with the socio-economic constraints such as limited resources and minimal access to veterinary or agricultural support, achieving immediate improvements in livestock management practices may not be practical. Nonetheless, the enhanced surveillance of cattle, facilitated by improved communication between livestock authorities and cattle owners during local outbreaks, can play a vital role in reducing exposure and preventing rabies infections.

The annual economic burden of rabies in Kazakhstan is estimated to be approximately USD 20.9 million, with an estimated USD 5.8 million allocated for managing rabies cases in animals [30]. A total of 183.172 individuals received PEP between 2009 and 2011. Most of these PEP administrations were associated with bites from stray dogs and wild animals [31]. According to the Global Alliance for Rabies Control, approximately 45,620 individuals receive PEP annually in Kazakhstan [8]. However, no information is available regarding PEP administration due to raw milk consumption and handling potentially sick cattle. Nevertheless, potential human exposure to rabies through the consumption of dairy products and contact with infected cattle has been documented in various regions worldwide [32,33]. Milk pasteurization effectively reduces the risk of rabies transmission and protects consumers from other milk-borne pathogens, including *Salmonella* spp. and *Brucella* species [34]. However, in certain regions, raw milk consumption remains the preferred choice due to its perceived superior taste and convenience [35]. In the present study, only 3% of respondents reported consuming raw milk, whereas the majority indicated either refraining from its consumption or subjecting it to boiling prior to ingestion. This finding contrasts with studies conducted in Zimbabwe and Tajikistan, where most participants preferred consuming raw milk [36,37].

Of the study population, only 7% of participants indicated that they would either process a carcass for sale or sell the entire carcass of cattle that had succumbed to disease. This could be explained by the fact that the disease-associated stigma and economic concerns often discourage farmers from reporting sick animals to veterinary authorities. The fear of financial losses may prompt livestock owners to sell or process carcasses rather than report an outbreak. In contrast, in India, nearly 90% of cattle owners did not prioritize proper carcass disposal [38], whereas the majority (87%) of participants in the present study reported following appropriate disposal practices, such as notifying veterinary authorities or burying the carcass.

In our study, participants reported engaging in on-farm health examination activities, including oral cavity examinations, wound dressing, assisting cattle during parturition, and handling sick animals, without utilizing basic PPE such as masks and gloves. Similar findings have been documented in studies conducted in Kazakhstan and Indonesia, where assisting animals during parturition without protective gloves was a common practice [39,40].

As rabies can be transmitted through cuts in the skin and mucous membrane contact with infected organs and body fluids, particularly saliva, involving in these activities poses a risk of exposure not only to rabies but also to other zoonotic diseases, such as brucellosis and leptospirosis. In Brazil, it was reported that veterinary staff contracted rabies while handling infected livestock, specifically cattle and goats [41]. Similarly, others reported an incident in Iran in which an animal health practitioner developed rabies after inserting unprotected, abraded hands into the oral cavity of a rabid cow [42]. Additionally, a case has been reported of a butcher acquiring rabies after handling an animal that had died from unspecified neurological symptoms [43,44]. However, proper hand hygiene, including washing hands with soap and water, can significantly reduce the likelihood of rabies exposure and the spread of other infectious diseases transmissible from animals to humans. In our study, 92% of participants who engaged in these practices reported washing their hands after handling animals. Consistent with findings from previous studies, our participants identified neighbors as the primary source of rabies-related information [28]. This can be attributed to the strong social networks in rural communities, where frequent interactions occur through collaborative agricultural activities and shared social events.

There are several limitations to this study that should be considered. The primary limitation of this study was the reliability of data collected from households. The farmers may have introduced bias by misreporting information, potentially exaggerating their losses. Additionally, recall bias among the farmers interviewed regarding rabies cases may have led to inconsistencies in reported events, making it challenging to establish causation with certainty. Considering these limitations, caution should be exercised when extrapolating our findings beyond the study areas. Nonetheless, this study offers valuable insights into the knowledge, attitudes, and practices of farmers concerning rabies in two regions of West Kazakhstan.

In developing countries such as Kazakhstan, it is recommended to implement targeted strategies, including training programs, to enhance public health awareness and preparedness among farmers and veterinary professionals for future outbreaks, including rabies. This can be effectively achieved through the dissemination of informational leaflets containing key epidemiological data, including prophylactic measures, early diagnostic methods, transmission pathways, and details regarding rabies sites. Leaflets incorporating visual aids, such as images and simplified text, offer the advantage of effectively communicating essential information to both literate and illiterate individuals. Moreover, strengthening community-based rabies education, integrating mandatory PrEP campaigns for high-risk livestock populations, and expanding government-supported PEP accessibility can significantly reduce the incidence of rabies. By incorporating these insights into national rabies control policies, this study underscores the urgency of a One Health approach, aligning human, animal, and environmental health sectors to enhance rabies surveillance, vaccination coverage, and outbreak response mechanisms. As rabies prevention and control strategies evolve, future research should focus on **longitudinal studies** to assess changes in livestock owners’ KAPs over time and evaluate the effectiveness of ongoing intervention programs. Additionally, exploring alternative strategies such as **community-driven rabies education, expansion of PrEP in livestock, and improved veterinary surveillance systems** will be crucial for refining control measures. Integrating these insights into national rabies policies could enhance long-term prevention efforts and ensure sustained progress toward rabies elimination in Kazakhstan.

## Figures and Tables

**Table 1:** Detailed socio-demographic characteristics of participants in both regions of Aktobe and Oral in West Kazakhstan (*n* = 688).

Variables	Categories	Aktobe (*n* = 344)	Oral (*n* = 344)	*p*-Value	Correlation Coefficient (r)
Age	25–44	120	76	0.33	−0.04
	45–64	126	156		
	Above 65	99	111		
Sex	Male	237	136	<0.02	0.20
	Female	107	208		
Education	Secondary	255	240	0.905	0.38
	Tertiary	62	70		
	Primary	27	34		
Occupation	Farmer	292	322	0.12	0.10
	Business	52	22		
Type of cattle	Exotic breeds	155	93	0.002	0.12
	Both breeds	189	251		
Number of cattle owned per household	More than 5	165	217	<0.025	0.15
	Less than 5	179	127		
Children attending school	Yes	113	268	<0.003	0.002
	No	231	76		
Dog ownership	Yes	196	212	0.060	0.08
	No	148	132		

**Table 2:** Summary of respondents with adequate knowledge of rabies.

Variables	Categories	Adequate Knowledge Yes/No	OR (95% CI)
Sex	Female	112/135	Ref. 0.97 (0.75–1.22)
	Male	223/145	
Education	Educated	83/192	Ref. 4.11 (3.46–5.33)
	Uneducated	168/172	
Residency	Aktobe	236/69	Ref. 5.14 (4.36–8.16)
	Oral	124/186	
Witnessed rabies	No	90/177	Ref. 3.43 (1.95–5.2)
	Yes	168/180	

OR = Odds Ratio. CI = Confidence interval.

**Table 3:** Summary of respondents with a favorable attitude toward rabies.

Variables	Categories	Favorable Attitude Yes/No	OR (95% CI)
Age	25–44	81/68	Ref. 0.77 (0.46–0.92) 0.84 (0.67–1.22)
	45–64	172/90	
	above 65	130/74	
Residency	Aktobe	157/110	Ref. 2.11 (1.86–4.66)
	Oral	227/121	
Witnessed rabies	No	175/137	Ref. 2.12 (1.05–3.84)
	Yes	210/93	

OR = Odds Ratio. CI = Confidence interval.

## Data Availability

The data are contained within this article and Supplementary Materials.

## Supplementary Materials

The following supporting information can be downloaded at: https://www.mdpi.com/article/10.3390/vetsci12040335/s1, Table S1: Questionnaire; Table S2: Raw data.
