# Supplementary material for: Smallholder Cattle Farmers’ Knowledge, Attitudes, and Practices Toward Rabies: A Regional Survey in Kazakhstan
Source: Vet Sci. 2025 Apr 4;12(4):335. doi: 10.3390/vetsci12040335 (PMC12030988; doi:10.3390/vetsci12040335)
Supplement: Supplementary file 1 [file vetsci-12-00335-s001.zip › vetsci-3537332-supplementary Table S1.pdf]

Survey- Questionnaire

1	What is your district of residence?	Yes/No
2	Please provide your age	
3	Please provide your gender	Male/Female
4	What is your education level?	Primary education<br>Secondary education<br>Tertiary level of education
5	What is your occupation?	Agriculture<br>Business
6	Livestock species on the farm	Cattle<br>Horse<br>Goat<br>Sheep
7	Where did you obtain information about anthrax?	Friends<br>Media<br>Neighbors<br>Veterinarian
8	Do you have knowledge about symptoms of animal rabies?	Yes/No
9	Do you have knowledge about symptoms of rabies?	Yes/No
10	Do you have children attending school?	Yes/No
11	Have you heard about rabies existence?	Yes/No
12	Have you participated in the Animal Health Training Program?	Yes/No
13	Have you participated in the Rabies Awareness Program?	Yes/No
14	How do you think a farmer can get rabies from animals (transmission route)?	By bite<br>By scratch<br>Consuming raw milk<br>Do not know<br>Handling infected animals<br>By wound contact<br>Other (specify)
15	Are you aware of human PEP?	Yes/No
16	Are you aware of animal PrEP?	Yes/No
17	What would you do if bitten by a rabid dog?	Visit Health Center<br>Do nothing<br>Wash bitten area

18	Do you think rabies can be preventable by vaccination?	Yes/No
19	Where do you obtain vaccine (source of vaccine)?	State veterinary service<br>Other
20	Have your animals been vaccinated against rabies?	Yes/No
21	Do you think rabies is a fatal disease?	Yes<br>No<br>Do not know
22	If a cow in your herd dies from suspected rabies, what would you do?	Report it to veterinary authorities and properly dispose of the carcass<br>Sell the carcass or use it for consumption<br>Bury the carcass without notifying authorities
23	Do you have a designated barn or shelter to house your cattle?	Yes, a well-structured barn with proper fencing and roofing<br>Yes, but it lacks proper fencing or roofing<br>No, my cattle are kept in an open area<br>No, my cattle roam freely without any designated shelter
24	Have you participated in rabies-affected animal slaughtering?	Yes/No

I have willingly participated in this interview. To the best of my knowledge and belief, all of the information given by me is correct and true.

Name of the interviewer: Signature:
